# Climatic drivers of the 2024 malaria outbreak in two neighbouring districts in northeastern India and its corridor: The indirect role of El Niño

**DOI:** 10.1016/j.onehlt.2026.101538

**Published:** 2026-08-19

**Authors:** Avik Kumar Sam, Ujjal Gogoi, Ananta Maji, Arup Borgohain, Rocky Pebam, Tarun Bhatnagar, Keshab Barman, Supriya Chaudhuri, Kalpana Baruah, Harish C. Phuleria, Ipsita Pal Bhowmick

**Affiliations:** a Environmental Science and Engineering Department, Indian Institute of Technology Bombay, Powai, Mumbai, India; b ICMR – National Institute of Health Research (NIHR), Dibrugarh, Assam, India; c District Health & Family Welfare Samiti, Alipurduar 736121, India; d Northeastern Space Applications Centre (NESAC), Department of Space, Government of India Umiam, Shillong, Meghalaya 793103, India; e ICMR – National Institute of Epidemiology (ICMR-NIE), Chennai, Tamil Nadu, India; f National Center for Vector Borne Diseases Control (NCVBDC), Govt of Assam, India; g Directorate of National Vector Borne Disease Control Programme, Ministry of Health and Family Welfare, Government of India, Delhi 110054, India; h Model Rural Health Research Unit (MRHRU), Department of Health Research, Government of India, Tripura, India.

**Keywords:** Malaria, Climate, El-Niño, Outbreak, Early-warning detection

## Abstract

**Background:**

Despite the progress made, malaria continues to impose a major global and regional burden. India contributes to almost half of the malaria cases in South-East Asia. In 2024, an unusual increase in malaria cases was observed in Alipurduar and neighbouring Kokrajhar district in Northeast India; regions that had maintained an API below 1 for several years. Thus, understanding the climatic and environmental factors of this outbreak is critical for early detection and prevention.

**Methods:**

Monthly epidemiological data on confirmed malaria cases in both districts were analysed from 2018 to 2024 along with meteorological variables and El Niño–Southern Oscillation indices. Multiple Poisson regression models incorporating lagged climatic parameters were developed separately and assessed using the RMSE for out-of-sample prediction performance.

**Results:**

The marked increase in malaria cases observed between August and December 2024 in both districts was primarily driven by *Plasmodium vivax* infections (∼95% cases in Alipurduar). The models showed no heteroscedasticity (*p* > 0.05) and captured the outbreak trend well in both districts. The lagged temperature was identified as a common significant predictor variable in both districts, with a delayed effect of 2–3 months. Precipitation was negatively associated with malaria in both districts, with a 1-month lagged impact in Alipurduar and none in Kokrajhar. The non-transferability of the models confirms a differential impact of weather variables, along with local vector dynamics, and an independent, indirect effect modification of El Niño conditions on the reported malaria cases in both districts.

**Conclusion:**

The lagged temperature increase associated with El Niño-driven climatic anomalies exerted a strong influence on the 2024 malaria outbreaks in Alipurduar and Kokrajhar. These results emphasise the role of integrating ENSO-based forecasting within malaria in early-warning systems. Early, climate-informed surveillance and planning for vector control would prevent localized resurgence and sustain malaria elimination gains, especially in low-API districts.

## Graphical abstract

**Figure:**
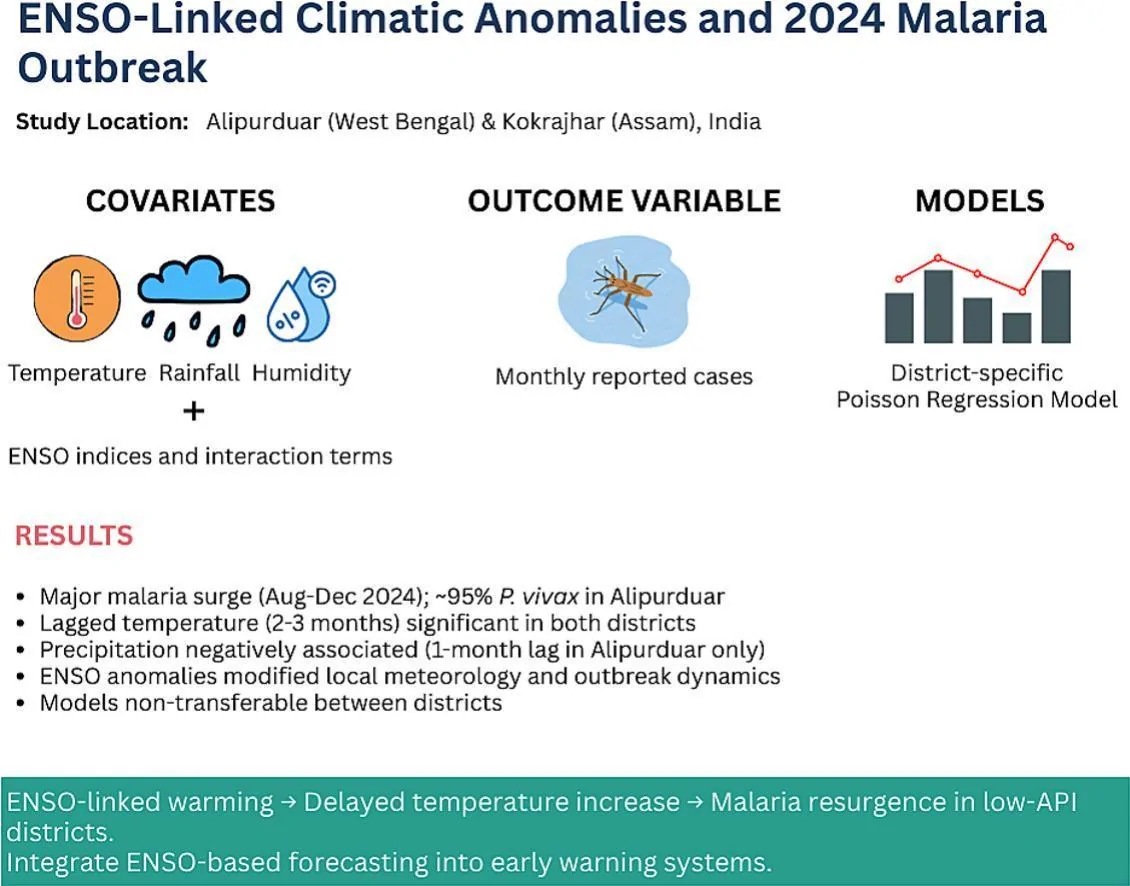

## 1 Introduction

Malaria remains a major global public health challenge, with an estimated 263 million cases and 597,000 deaths reported worldwide in 2023 [36]. The Global Burden of Disease study attributed ∼51 million Disability-Adjusted Life Years (DALYs) to malaria, reflecting substantial losses due to premature mortality and morbidity in 2023 [11]. Despite substantial progress in malaria control, India continues to contribute a significant proportion of the malaria burden in the WHO Southeast Asia Region, reporting 227,564 cases and 83 deaths in 2023 and accounting for nearly half of all regional cases [22]. Furthermore, disruptions to malaria prevention, diagnosis, and treatment services during the COVID-19 pandemic resulted in an estimated 13 million additional malaria cases and 63,000 excess deaths globally during 2020–2021, underscoring the fragility of recent malaria control gains [35].

Although malaria transmission has declined substantially in many parts of India, outbreaks in low-transmission or traditionally non-endemic settings remain a major public health concern because they can rapidly escalate local disease burden, overwhelm health systems, and reverse elimination progress [29], [30]. Such outbreaks are often driven by a complex interplay of environmental, entomological, climatic, and health-system-related factors [19]. In particular, climatic variability can strongly influence malaria transmission through its effects on mosquito breeding, vector survival, biting behaviour, and parasite development [26], [32]. Temperature, precipitation, and relative humidity directly affect vector abundance and the extrinsic incubation period of *Plasmodium* parasites, while large-scale climatic phenomena such as the El Niño Southern Oscillation (ENSO) can alter regional precipitation and temperature patterns over prolonged periods [14], [18], [33]. Previous studies have demonstrated associations between ENSO variability and malaria transmission dynamics across several endemic regions, including India [3], [5], [6]. However, evidence from northeastern India, particularly from low-transmission districts experiencing localized outbreaks, remains limited.

Alipurduar District, located in the northern part of West Bengal, lies along the strategically important “chicken neck” corridor connecting northeastern India with the rest of the country and neighbouring Bhutan, making it epidemiologically important for vector-borne disease transmission. Although the district maintained an Annual Parasite Incidence (API) below 1 between 2017 and 2022, localized outbreaks continued to occur in specific areas, including Kumargram and Turturi Primary Health Centres (PHCs), as well as in the adjacent Kokrajhar District of Assam [2]. Notably, a malaria outbreak was reported in 2019 in the Raydak Tea Estate-1 Sub-Centre under Turturi PHC [31], followed by a *Plasmodium vivax* outbreak in Saralpara, Kokrajhar, in 2022 [2]. Entomological investigations in the region identified competent malaria vectors, including *Anopheles annularis*, *An. maculatus*, and *An. culicifacies* [28].

A substantial increase in malaria cases was again observed in Alipurduar between August and December 2024, particularly in the Kumargram and Kalchini blocks, with case numbers considerably exceeding those reported during the previous four years (Unpublished Data). Interestingly, the outbreak was characterized by a marked predominance of *P. vivax*, with the proportion of *P. vivax* cases in 2024 approximately 95% higher than in 2022 and 2023, suggesting a potential shift in parasite dynamics. During the same period, neighbouring Kokrajhar District in Assam also reported increased malaria transmission, particularly in the Kachugaon, Dotma, and Balajan PHCs (Unpublished Data). Although Kokrajhar has historically been considered a low-transmission district, the region possesses environmental conditions favourable for malaria transmission, including prolonged precipitation, abundant mosquito breeding habitats, optimal temperature and humidity, and ecological suitability for parasite development, facilitating sustained vector proliferation throughout the year (Unpublished Data).

Field investigations conducted during the Alipurduar outbreak identified forest exposure as an important risk factor for malaria infection (Unpublished Data). However, these ecological conditions have remained relatively stable over time, and no major land-use or environmental changes were identified that could independently explain the sudden rise in malaria incidence in 2024. Moreover, because both Alipurduar and Kokrajhar consistently reported API values below 1, these areas were not prioritized for large-scale vector control interventions such as long-lasting insecticidal nets (LLINs) or indoor residual spraying (IRS) under the national vector-borne disease control programme. In this context, climatic variability may have contributed to increased vector abundance, altered transmission suitability, or expanded transmission windows, thereby facilitating the observed outbreaks in the two neighbouring districts.

In the present study, we investigate the role of climatic variability, including delayed effects of meteorological conditions and broader climatic influences, in shaping the recent malaria outbreaks in Alipurduar and Kokrajhar, two neighbouring districts in northeast India. As secondary objectives, we characterize the temporal distribution and magnitude of malaria cases in the two districts.

## 2 Methods

### 2.1 Study area description

**Fig. 1:**
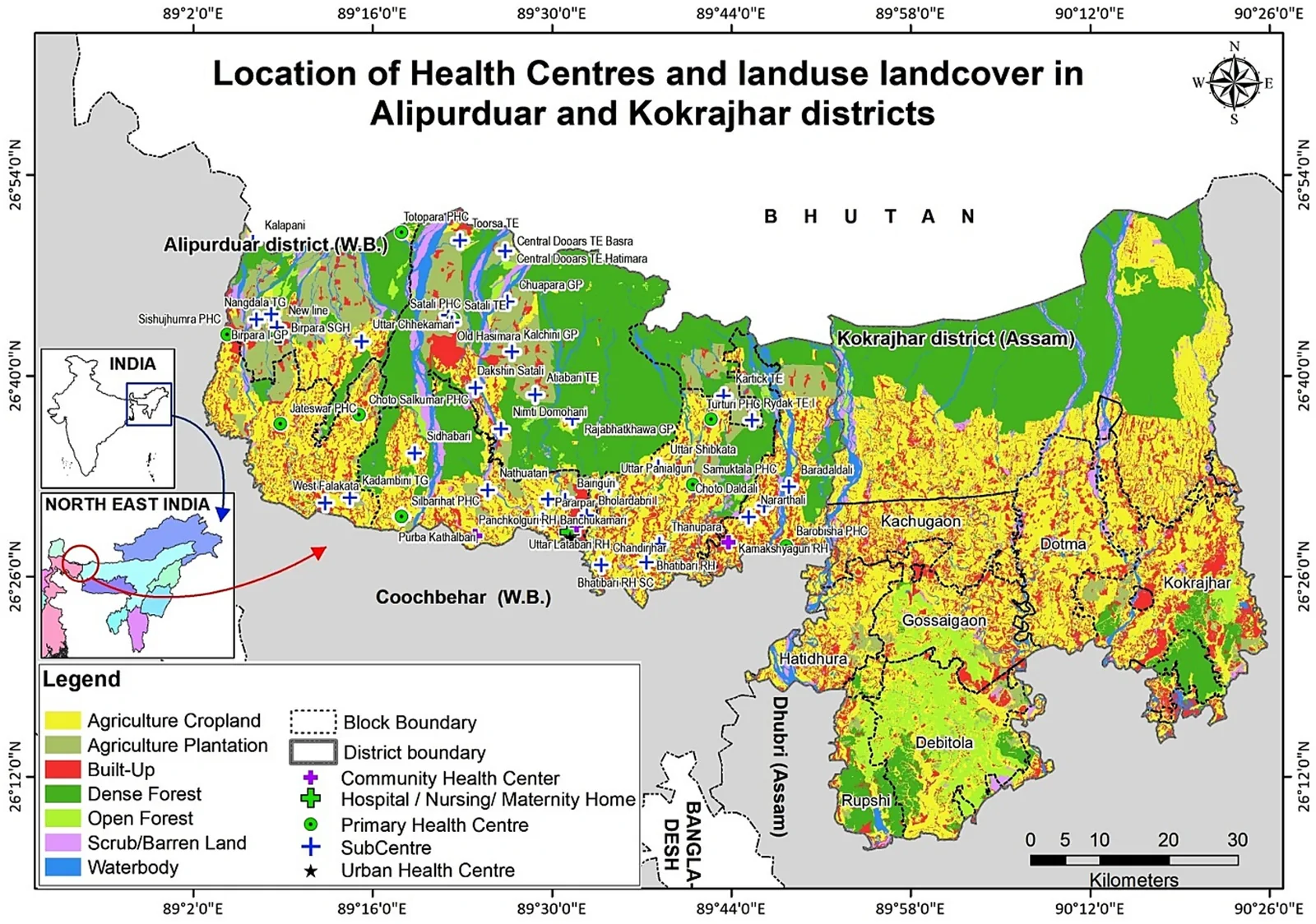
Location of Health Centres and land-use land-cover in Alipurduar and Kokrajhar districts.

Alipurduar district of West Bengal has gained recognition as an important link in the “chicken neck” corridor, connecting the North-eastern region of India to the rest of the country and shares an international boundary with Bhutan. The region is divided into two-thirds flat plains to the south and approximately 4% elevated (>900 m above MSL) territory towards the northern international boundary. The average yearly precipitation is less in the south (less than 250 cm), more in the north (more than 300 cm), and less in the north at high elevations (less than 150 cm). Approximately 40% of the area in Alipurduar is covered by forests, followed by agricultural land and tea gardens, with rural and semi-urban settlements occupying the remaining area. Kokrajhar is a district in Assam that shares the international border with Bhutan in the north and the national border with Alipurduar, West Bengal, in the west. About 29% of the district was covered by forests, while the average annual precipitation varied between 170 and 200 cm. The study location is visually represented in Fig. 1, which also shows the location of health centres and the underlying land-use land-cover patterns.

The Land use and land cover maps were prepared using IRS P5 Cartosat-1 (2.5 m) and IRS Resources-2 LISS IV (5.8 m) satellite data of 2018–2024 at 1:10,000 scale, UTM Projection WGS 84 datum following ISRO/DOS classification standards using visual interpretation techniques.

### 2.2 Malaria data collection

Month-wise malaria case data, including the number of individuals tested, species-wise positive cases, and cases treated, were collected from the respective district malaria programs for Alipurduar (January 2018–December 2024) and Kokrajhar (January 2019–December 2024). Because the population did not change according to the official estimates, the monthly incidence rates in our study were the same as the number of cases reported each month.

### 2.3 Meteorological data

We obtained the monthly data on Relative Humidity (RH, %), Temperature (Temp, °C) and Precipitation (Prec., mm) from the ERA5 reanalysis dataset, which is produced by the European Centre for Medium-Range Weather Forecasts through the Copernicus Climate Change Service [10].

The anomalies in monthly sea surface temperature observed in the Niño 3.4 region between 2017 and 2024, defined here as the El Niño Southern Oscillation or ENSO, were sourced from the NOAA Climate Prediction Centre [24].

a. **Bias correction**

We corrected the meteorological data using locally sourced weather station data from Alipurduar, which was available for a year, as satellite-derived precipitation estimates may exhibit systematic bias relative to ground-based weather stations.

For temperature and relative humidity, we fitted a linear regression model between the two sources. The equations obtained were:

$$RH_{\mathrm{satellite}}=0.8433\times RH_{\mathrm{station}}+21.61,R^{2}=0.98$$

$$\mathrm{Tem}p_{\mathrm{satellite}}=1.0065\times \mathrm{Tem}p_{\mathrm{station}}-1.5418,R^{2}=0.985$$

These equations were used to estimate the corrected % Relative Humidity and mm Precipitation. For precipitation, we used the multiplicative bias correction factor to obtain the corrected precipitation [12], as provided below,

$$\mathrm{Pre}c_{\mathrm{Corrected}}=\mathrm{Pre}c_{\mathrm{satellite}}\times \frac{\sum \mathrm{Pre}c_{\mathrm{station}}}{\sum \mathrm{Pre}c_{\mathrm{satellite}}}$$

Similarly, corrections were applied to the Kokrajhar data.

b. **Lag creation**

To assess the delayed impact of weather variables and ENSO, we developed lagged components for each by shifting the variables back. For relative humidity, temperature, and precipitation, lag periods of 0–6 months were considered to capture the short-term delayed effects of local climatic conditions on mosquito breeding, vector survival, and parasite development, as reported in previous malaria–climate studies, while ENSO lags of 0–12 months were evaluated to account for the longer-term influence of large-scale climate variability on malaria transmission dynamics [7], [20], [25].

In the present study, weather and ENSO data were collated one year prior to the malaria case data period to facilitate the construction of lagged variables. This ensured that, after applying lag structures and removing observations with missing values generated during the lagging process, sufficient malaria case data were retained for analysis.

c. **Data Processing**

Prior to modelling, we investigated the temporal effects of ENSO on meteorological conditions observed in Alipurduar and Kokrajhar using the Granger causality test. Granger causality is a statistical time-series approach used to determine whether past values of one variable improve the prediction of another variable beyond the information contained in its own past values. In this study, the test was applied to assess whether lagged ENSO anomalies improved the prediction of local meteorological variables, including temperature, precipitation, and relative humidity. The null hypothesis assumes that past ENSO values do not provide additional predictive information for the meteorological variable under consideration. Rejection of the null hypothesis (*p*-value <0.05) was interpreted as evidence of a statistically significant predictive temporal association. We performed Granger causality tests with lag periods ranging from 1 to 12 months, consistent with the temporal scale of seasonal ENSO-related climate variability [38]. Previous studies have also reported associations between ENSO variability and wet and dry conditions in India [8]. However, we note that Granger causality reflects predictive relationships within time-series data and should not be interpreted as evidence of direct physical causation.

In Alipurduar, we observed a statistically significant association (*p*-value <0.05) with % relative humidity (lags of 8–11 months) and with lagged temperature (8-month lag). No such relationship is observed with the precipitation (mm). In contrast, a significant association (*p*-value <0.05) exists between temperature and the ENSO anomaly in Kokrajhar at lags of 8–12 months. Further, based on the Granger causality test, we observed a statistically significant association between the ENSO anomaly and precipitation at a 9-month lag. However, no significant associations were observed between ENSO anomaly and % Relative Humidity in Kokrajhar. Based on the findings, we used the lagged terms of weather variables directly in our model, while the interaction term between ENSO and the weather variables was used to estimate the effect modification by ENSO, obtained by multiplying the two values. ENSO anomalies were not included as direct predictors in the regression models because their influence on malaria transmission is primarily mediated through local meteorological conditions, including temperature, precipitation, and relative humidity [1], [15]. Accordingly, locally observed weather variables were retained as the primary predictors to evaluate the effects of proximal climatic drivers and potential effect modification by El Niño. To assess potential multicollinearity among ENSO anomalies and meteorological variables, we calculated Variance Inflation Factors (VIFs). All predictor variables showed low multicollinearity (VIF < 5), indicating acceptable independence among the climatic covariates.

### 2.4 Model structure

Following this, we used a Poisson regression model with monthly cases as the response variable and lagged meteorological variables as independent variables. We used data up to 2023 for training the model and validated it using 2024 data. The model assumes that the number of reported malaria cases $Y_{i}\;$for $i^{\mathrm{th}}$observation follows a Poisson distribution with the mean expected value of $\lambda_{i}$:

$$Y_{i}∼\mathrm{Poisson}\;\left(\lambda_{i}\right)$$

$$\log\left(\lambda \right)=\beta_{0}+\beta_{1}\times \text{Precipitation}\;{\left(\mathrm{mm}\right)}_{i}+\beta_{2}\times \text{Relative Humidity}\;{\left(\%\right)}_{j}+\beta_{3}\times \text{Temperature}\;{\left({}^{\circ}C\right)}_{k}+\beta_{4}\times {\text{Prec}}_{i}\times {\text{ENSO}}_{l}+\beta_{5}\times {\text{Temp}}_{k}\times {\text{ENSO}}_{l}+\beta_{6}\times {\mathrm{RH}}_{j}\times {\text{ENSO}}_{l}++\beta_{7}\times \text{Year}+\epsilon$$

where *i*, *j* and *k* denote the different combinations of lags between 0 and 6 months for Precipitation, Relative Humidity and Temperature, respectively, and *l* denotes the lag between 0 and 12 months for ENSO anomaly. Hence, β_1_, β_2_ and β_3_ represent the magnitudes of the delayed effects of precipitation, RH and temperature on dengue cases at *i*, *j* and *k* lags, respectively. The coefficients, β_4_, β_5_ and β_6,_ represent the magnitudes of the interaction terms discussed above. Further, a “Year” variable was included in the analysis to account for temporal autocorrelation and interannual variability in malaria incidence that may not be fully captured by the climatic covariates. The variable represented the corresponding calendar year for each observation and was included as a temporal control term in the model.

Multiple candidate Poisson regression models were developed using different combinations of lagged climatic variables. Model performance was jointly evaluated using Akaike Information Criterion (AIC), Bayesian Information Criterion (BIC), coefficient of determination (R [2]), and Root Mean Square Error (RMSE) calculated on the test dataset. Models with lower AIC, BIC, and RMSE values and higher R^2^ values were considered to provide improved model fit and predictive performance. More information about these parameters can be found here [13]. The final model was selected based on the overall consistency of these evaluation metrics. To minimize overfitting, lag ranges were restricted to biologically plausible periods reported in previous malaria–climate studies as discussed in Section 2.3, and model generalizability was evaluated using the validation dataset (2024). Simultaneously, we developed Negative Binomial models separately to explain the outbreaks in both districts, but the RMSE was significantly higher than that obtained for the Poisson model. We also performed the White-Het test for heteroscedasticity to determine whether the error variance is constant, while the normality of the residuals was also assessed using the visual diagnostic plots.

Further, to evaluate the robustness, additional sensitivity analyses were conducted in which ENSO was included as a main effect alongside the ENSO × meteorological interaction terms. Models with and without ENSO main effects were compared using RMSE, AIC, and BIC criteria, and the resulting lag structures and effect estimates were evaluated to determine whether inclusion of ENSO as an independent predictor altered the interpretation of the climate–malaria associations.

## 3 Results

### 3.1 Temporal trend in reported malaria cases and weather variations

In Alipurduar district, 31 and 53 individuals tested positive for malaria in 2021 and 2022, respectively; this number declined to 41 in 2023 before increasing to 744 in 2024. In Kokrajhar, the number of malaria-positive individuals increased from 32 in 2021 to 95 in 2022 and further rose to 256 in 2023 and 3808 in 2024. Overall, the number of cases in both Alipurduar and Kokrajhar was relatively higher between September and November (Fig. 1a & 1b). This seasonal pattern was further supported by a Kruskal–Wallis test, which demonstrated significant monthly variation in malaria incidence reported (Alipurduar: H = 30.4, *p* < 0.01; Kokrajhar: H = 33.8, *p*-value <0.01), indicating a pronounced transmission peak during the September–November period. Potential environmental and occupational factors, such as the presence of slow-moving streams and rivers originating from Bhutan and increased outdoor exposure during mustard cultivation activities, may contribute to the prolonged transmission season; however, these factors were not directly evaluated in the present study.

The detailed table on Blood Smear Collection (BSC), Blood Smear Examination (BSE), number of cases with *Plasmodium falciparum* (Pf), number of cases with *Plasmodium vivax* (Pv) and Annual Parasite Incidence (API) is provided in the supplementary (Table S1). India has maintained an API below 1 since 2012 and reported a national API of around 0.18 in 2024 [23]. Compared to the long-term declining malaria trend in India, both Kokrajhar and Alipurduar showed relatively low transmission during 2020–2023, with API < 3 since 2020 (Table S1). However, Kokrajhar reported a markedly higher API of 35.9 in 2024, while Alipurduar reported an API of 4.5 (Table S1), indicating a substantial localized resurgence compared to both recent district-level trends and the overall national malaria situation in India.

The variations in the weather variables (temperature (°C), relative humidity (%), precipitation (mm) and the ENSO anomaly (represented by the El-Niño anomaly) in Alipurduar and Kokrajhar are shown in the supplementary as Figs. S2 and S3, respectively. We observe similar temperature profiles over the years. The highest precipitation was observed during the monsoon season, particularly between June and August, in both Alipurduar and Kokrajhar. Mean monthly precipitation during the monsoon exceeded 250 mm in both districts, with peak cumulative monthly precipitation reaching ∼545 mm in Alipurduar and ∼ 552 mm in Kokrajhar. In contrast, precipitation between September–November was substantially lower, with mean monthly precipitation of ∼45 mm in both districts. Relative humidity remained consistently high throughout the year, particularly during the monsoon season, with mean humidity levels exceeding 92% in both districts, while humidity during the peak transmission period remained above 84%. Between May 2020 and April 2023, negative ENSO anomalies were observed. However, positive anomalies were reported between May 2023 and June 2023, peaking in December 2023 and the following January 2024.

### 3.2 Inferences from the model

Based on the out-of-sample predictive performance of the models against the test data, the optimal combination of lags was selected. The minimum RMSEs obtained from models separately explaining the 2024 outbreaks in Alipurduar and Kokrajhar were 12.9 and 26.1, respectively, indicating a differential delayed impact of weather variables on the reported malaria cases in each district. The selected final models, along with the magnitude of dependence for each variable, are provided in Table 1.

**Table 1:** Marginal effects observed for the meteorological variables in both Alipurduar and Kokrajhar.

District	Variable	Lag	Marginal Effect (95% CI)
Alipurduar	Relative Humidity (%)	2	**0.04 (0.03, 0.06)**
	Temperature (°C)	2	**0.2 (0.16, 0.24)**
	Precipitation (mm)	1	**−0.003 (−0.003, −0.002)**
	Year	NA	**−0.28 (−0.35, −0.21)**
	ENSO × RH	12, 2	**−0.07 (−0.09, −0.05)**
	ENSO × Temperature	12, 2	**0.28 (0.21, 0.36)**
	ENSO × Precipitation	12, 1	**−0.0007 (−0.002, −0.001)**
Kokrajhar	Relative Humidity (%)	4	**−0.03 (−0.04, −0.013)**
	Temperature (°C)	3	**0.33 (0.30, 0.37)**
	Precipitation (mm)	0	**−0.0015 (−0.002, −0.001)**
	Year	NA	**0.29 (0.22, 0.37)**
	ENSO × RH	12, 4	−0.004 (−0.017, 0.008)
	ENSO × Temperature	12, 3	**0.055 (0.011, 0.098)**
	ENSO × Precipitation	12, 0	**−0.002 (−0.003, −0.001)**

The statistically significant effects (*p*-value <0.05) are highlighted in bold. NA: Not applicable.

**Fig. 2:**
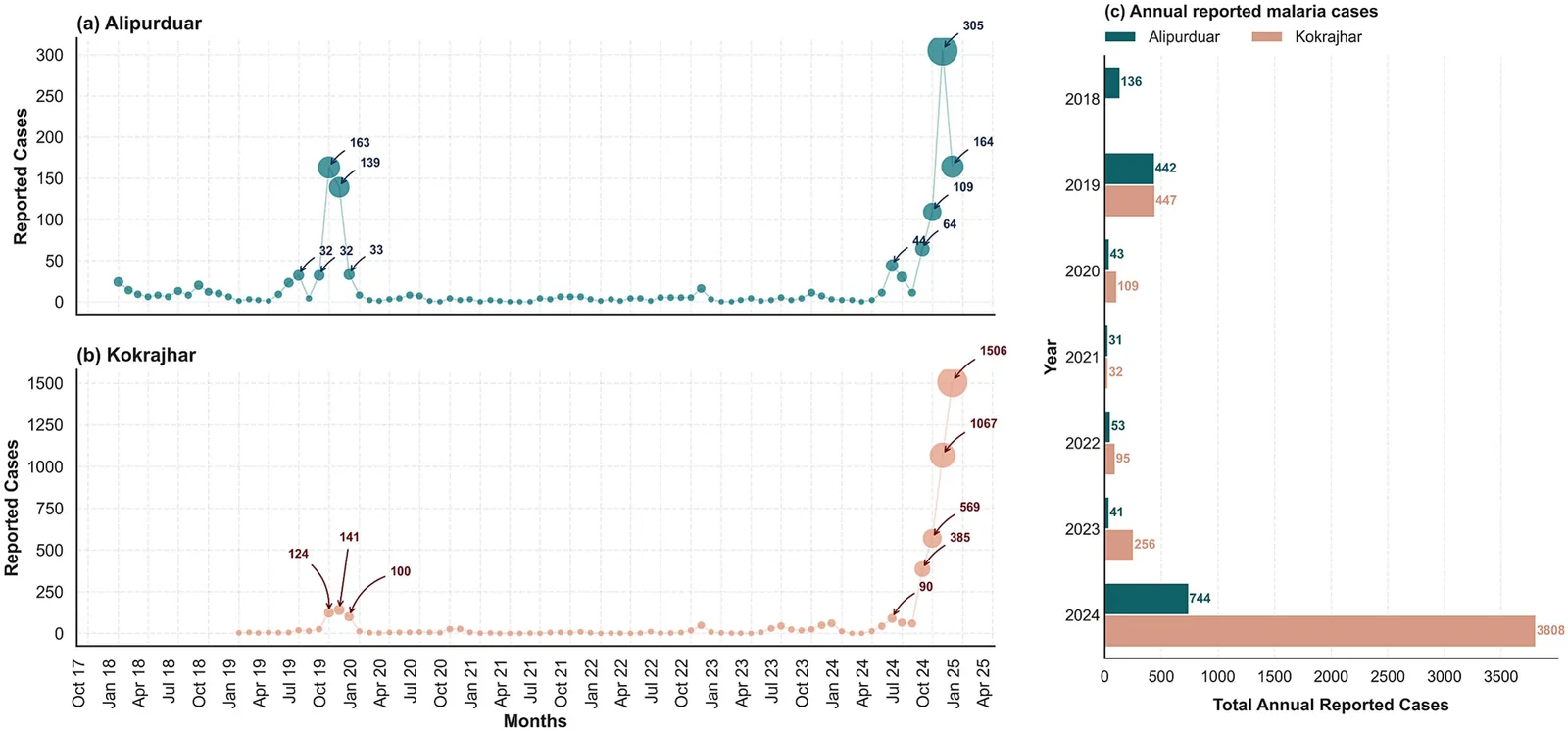
Month-wise reported malaria cases from 2017 to 2024 in (a) Alipurduar, and (b) Kokrajhar district. (c) Annual reported cases in Alipurduar and Kokrajhar.

We note that the malaria cases are positively associated with relative humidity and temperature observed two months ago. However, the interaction term between ENSO and lagged relative humidity was negatively associated, suggesting a marginal effect: with increasing ENSO anomalies, relative humidity had a negative impact on outbreaks. Conversely, the overall impact of temperature became increasingly pronounced with increasing ENSO, as indicated by a stronger positive dependence. Previously, an increase in temperature was linked to a reduction in the Extrinsic Incubation Period in Chennai [16], thus increasing the transmission risk of malaria. The comparison between the actual and predicted monthly cases for Alipurduar in the training and test datasets is shown in Fig. 2a and b, respectively.

**Fig. 3:**
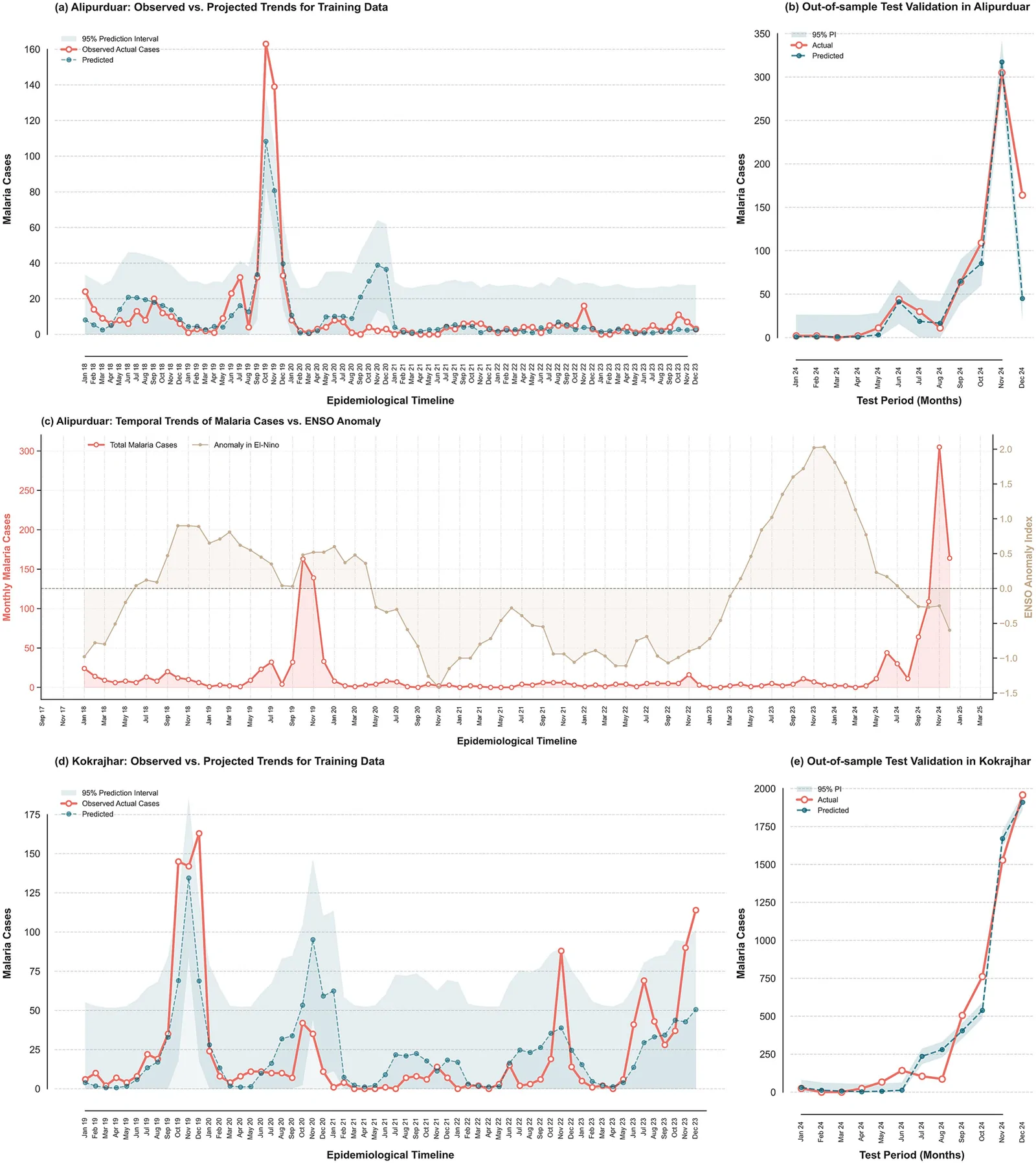
Comparison between actual and predicted malaria cases in Alipurduar for (a) training and (b) test datasets. (c) Variation in ENSO anomaly and the reported malaria cases in Alipurduar. Comparison between actual and predicted malaria cases in Kokrajhar for (d) training and (e) test dataset.

The potential effect of the 12-month lagged ENSO anomaly can also be observed in Fig. 3c, where increased malaria cases in Alipurduar during November 2024 occurred approximately 12 months after the highest positive ENSO anomaly. These findings suggest a possible temporal association between ENSO variability and the 2024 malaria outbreak in Alipurduar, potentially mediated through local meteorological conditions. The observed relationship was supported by the model performance (observed R^2^ = 0.84); however, the results should be interpreted as predictive statistical associations rather than evidence of direct causality.

In Kokrajhar, we observed different delayed impacts of weather variables on the malaria outbreaks, compared to Alipurduar (Table 1). We note that there is a negative association between cases with 4-month lagged relative humidity and present precipitation. However, the 3-month lagged temperature is positively associated with the cases. There is also a positive dependence on the variable year. Additionally, the limiting role of both 4-month-lagged relative humidity and precipitation is reduced by their interaction with 12-month-delayed ENSO anomalies. For temperature, there is a relative decrease in the magnitude of association with ENSO, although it still has a positive effect. The comparison between the observed and predicted cases for the training and test datasets in Kokrajhar is shown in Fig. 3d and e, respectively, and indicates robust prediction of 2024 outbreaks (observed R^2^ = 0.97).

We also observed that in Alipurduar, increased precipitation was associated with a small but significant decrease in cases at a 1-month lag, although the effect size was considerably smaller than that of temperature. This likely suggests a potential role of high precipitation in larval washing and a reduction in viable breeding sites [27], as we also observed relatively fewer cases during months with high precipitation. The significant negative ENSO × precipitation interaction at a 12-month ENSO lag suggests that ENSO conditions from the previous year modify the effect of precipitation over time. In Kokrajhar, precipitation showed an immediate negative association with cases (lag 0), while the stronger negative ENSO × precipitation interaction at a 12-month ENSO lag indicates a delayed climatic influence, whereby past ENSO variability alters the current impact of rainfall on cases. However, these associations should be interpreted cautiously, as the observed effect sizes were relatively small and may also reflect the influence of other unmeasured environmental or socio-ecological factors.

We note that sensitivity analyses including ENSO main effects produced district-specific results. In Alipurduar, the ENSO main effect was significantly associated with malaria incidence and marginally improved model performance (RMSE = 12.3), whereas in Kokrajhar the ENSO main effect was not statistically significant and did not improve model performance. Inclusion of the ENSO main effect also resulted in changes to the optimal lag structure selected for several meteorological variables based on RMSE, AIC, and BIC criteria, suggesting shared climatic information between ENSO and local weather conditions. Nevertheless, the overall interpretation of the associations between meteorological variables, ENSO-related effect modification, and malaria incidence remained broadly consistent across model specifications*.*

## 4 Discussion

Temperature has been established as a significant factor in the transmission dynamics and distribution of malaria, influencing both the *Anopheles* vector and the *Plasmodium* parasite [25], [34], [39], [40]. There is a delayed impact arising from the time taken for the mosquito to develop, the extrinsic incubation period, and the incubation period in humans, which further delays the onset of symptoms and, consequently, malaria diagnosis. Previously, a 1–4 month lag in the impact of climatic variables on malaria transmission was identified in rural Gwanda, Zimbabwe, with a peak association observed for ∼3 months [7]. Multiple studies have estimated a broad range of optimal temperatures between 25° and 33 °C for malaria transmission [4], [17], [21], [26]. In both Alipurduar and Kokrajhar, the monthly-averaged temperature ranged from 20° to 30 °C (Figs. S2, S3). At higher temperatures within the optimum window, mosquito larval development, biting rates, and parasite maturation rates are often accelerated [4], [25], and precipitation also influences breeding success and vector habitat availability [21].

Further, ENSO conditions observed 12 months back were moderately associated (*r* = 0.5) with malaria incidences in Venezuela [20]. A delayed impact of ENSO events on malaria transmission was also observed in Colombia, Guyana, Peru [3], [6], French Guiana [9], and Ethiopia [37]. Thus, a real-time early warning detection system should be developed based on El Niño conditions to ensure adequate preparedness and response, thereby reducing the likelihood of any potential outbreak. Previous calls to implement intervention measures in line with ENSO events [5] are reiterated, as better planning for allocating interventions can help prevent outbreaks.

Overall, the selected models showed no significant heteroscedasticity (White heteroscedasticity test, *p*-value >0.05) and were able to capture the broad temporal trends of the 2024 malaria outbreaks within acceptable predictive ranges. The findings suggest significant temporal associations between ENSO variability, local meteorological conditions, and malaria incidence in both Alipurduar and Kokrajhar. In particular, the significant ENSO – temperature, ENSO - precipitation, and ENSO - relative humidity interaction effects suggest the relationship between local weather conditions and malaria incidence is modulated by ENSO phase. This pattern is consistent with the hypothesis that ENSO acts as a large-scale climatic driver that modifies local environmental conditions conducive to malaria transmission [1], [15]. Changes in temperature, precipitation, and humidity can influence mosquito breeding habitat availability, vector survival, biting behaviour, and the rate of *Plasmodium* development within the mosquito vector, thereby altering transmission potential. These findings therefore support a climate-mediated pathway linking ENSO variability to malaria risk through modification of local meteorological effects. However, because ecological and entomological variables were not directly measured, the present analyses identify predictive temporal associations rather than direct causal mechanisms.

The study also identified significant delayed effects of meteorological variables, particularly lagged temperature, on malaria incidence in both districts. Such lagged associations are biologically plausible because climatic conditions influence several sequential stages of malaria transmission, including larval habitat formation, mosquito population growth, adult vector survival, and the extrinsic incubation period of *Plasmodium* parasites [4], [21], [25]. These ecological and biological processes often operate over periods of weeks to months, resulting in delayed effects of climatic variability on malaria incidence. Similar lagged temperature effects have been reported in previous malaria–climate studies conducted in tropical and subtropical regions.

Interestingly, although Alipurduar and Kokrajhar are geographically adjacent districts with broadly similar climatic conditions, the predictive performance of models trained in one district was reduced when applied to the other district. However, this should not be interpreted as evidence of fundamentally different transmission mechanisms. Instead, the observed differences in predictive performance may reflect local heterogeneity in vector ecology, environmental conditions, healthcare access, surveillance practices, case distributions, intervention coverage, and other unmeasured ecological or socioeconomic factors not included in the present analysis. Thus, our findings highlight the importance of considering local-scale variability when developing climate-informed malaria prediction models.

### 4.1 Limitations of the study

Our study has few limitations that should be considered while interpreting the findings. First, the analyses relied on ERA reanalysis data at a spatial resolution of 0.25° × 0.25°, which may not fully capture local-scale precipitation variability; future studies could benefit from incorporating higher-resolution station-based gridded datasets, such as Indian Meteorological Department (IMD) precipitation products, and applying bias-correction approaches where appropriate. Second, the bias-correction equations derived using observational weather data from Alipurduar were also applied to Kokrajhar because comparable station-based data were unavailable for the latter. Although the two neighbouring districts share broadly similar climatic and ecological characteristics, the use of common correction equations may introduce uncertainty arising from local-scale climatic and measurement differences. Thus, future studies should incorporate district-specific observational datasets to improve calibration accuracy and reduce potential bias.

Third, the analysis primarily focused on climatic variables and did not account for other determinants of malaria transmission, including vector density, land-use patterns, human mobility, socioeconomic conditions, healthcare access, and vector control interventions. While major changes in these factors were unlikely during the study period in the low-endemic study districts, residual confounding from unmeasured ecological and socio-environmental factors cannot be ruled out. Fourth, the analyses were conducted using aggregated district-level data and are therefore subject to potential ecological fallacy; consequently, the observed associations should not be interpreted as representing individual-level malaria risk. Fifth, while ENSO-related climatic variability showed significant associations with malaria incidence, the mechanistic pathways underlying these relationships were not directly tested and remain indirect. Therefore, future studies should integrate higher-resolution climatic datasets with entomological, environmental, demographic, and intervention-related data to better characterize the complex drivers of malaria transmission in low-endemic settings. Longitudinal studies incorporating vector surveillance, land-use dynamics, human mobility, and climate variability, including large-scale climatic phenomena such as ENSO, may further improve outbreak prediction and support the development of climate-informed malaria early warning systems in northeastern India. Finally, the study was conducted in two neighbouring districts of northeastern India. Therefore, caution is warranted when extrapolating these findings to other regions of India, where climatic conditions, malaria epidemiology, vector ecology, and health system characteristics may differ.

## 5 Conclusion

In conclusion, the present study demonstrates significant temporal associations between ENSO variability, local meteorological conditions, and malaria incidence in Alipurduar and Kokrajhar, highlighting the potential utility of climate-informed early warning systems for outbreak preparedness in historically low-transmission settings. The observed lagged effects of temperature and other climatic variables are biologically plausible and consistent with established malaria transmission dynamics involving vector development, survival, and parasite maturation. The prolonged transmission period and predominance of *Plasmodium vivax* during the 2024 outbreaks further suggest that favourable post-monsoon environmental conditions may contribute to localized outbreaks even in areas with relatively low routine intervention prioritization. At the same time, the reduced transferability of predictive models between neighbouring districts emphasizes the importance of local ecological and epidemiological heterogeneity in shaping malaria dynamics. Although the study identifies predictive temporal relationships rather than direct causal mechanisms, the findings support the integration of climatic indicators, including ENSO signals, into region-specific malaria surveillance and preparedness strategies, while underscoring the need for further multidisciplinary investigations into vector ecology, environmental drivers, and intervention effectiveness. However, because the analyses were conducted using aggregated district-level data and did not include individual-level exposure or entomological measurements, the observed associations should be interpreted as population-level patterns rather than direct evidence of individual-level malaria risk or underlying transmission mechanisms.

## CRediT authorship contribution statement

**Avik Kumar Sam:** Writing – original draft, Visualization, Software, Methodology, Formal analysis. **Ujjal Gogoi:** Writing – review & editing, Writing – original draft, Visualization, Software, Formal analysis. **Ananta Maji:** Investigation, Data curation. **Arup Borgohain:** Validation, Software, Data curation. **Rocky Pebam:** Visualization, Validation, Software, Data curation. **Tarun Bhatnagar:** Writing – review & editing, Validation. **Keshab Barman:** Investigation, Data curation. **Supriya Chaudhuri:** Investigation, Data curation. **Kalpana Baruah:** Writing – review & editing, Validation. **Harish C. Phuleria:** Writing – review & editing, Validation, Supervision, Resources, Methodology, Conceptualization. **Ipsita Pal Bhowmick:** Writing – review & editing, Visualization, Validation, Supervision, Resources, Project administration, Methodology, Investigation, Funding acquisition, Conceptualization.

## Ethics declaration

Written informed consent to take part in the study and to publish the article has been obtained from all participants or their legal representatives. The privacy rights of participants have been observed.

This study was performed in compliance with relevant laws, regulatory frameworks and guidelines where the research took place.

This study was approved by the Institutional Ethics Committee (IEC)/Institutional Review Board (IRB), ICMR RMRC NE, Dibrugarh. (Approval No. RMRC/Dib/IEC (Human)2020-21/293)

Ethical permission for the work has been obtained by the Institutional Bioethics Committee. The study protocol was approved by the Institutional Ethics Committee (IEC)/Institutional Review Board (IRB), ICMR RMRC NE, Dibrugarh on 02.11.2021 [RMRC/Dib/IEC (Human)2020-21/293], which is according to the Declaration of Helsinki.

## Funding

The study was conducted as part of a project funded by the 10.13039/501100001411Indian Council of Medical Research (ICMR) extramural grant no, NER/78/2020-ECD-I (Extramural Ad-hoc (icmr.org.in). The funders had no role in the study design, data collection, analysis, and preparation of the manuscript.

## Declaration of competing interest

The authors declare that they have no known competing financial interests or personal relationships that could have appeared to influence the work reported in this paper.

## Data Availability

The data presented in this study are available on request from the corresponding author. The data are not publicly available due to ethical and privacy reasons.
