# Supplementary material for: Climatic drivers of the 2024 malaria outbreak in two neighbouring districts in northeastern India and its corridor: The indirect role of El Niño
Source: One Health. 2026 Aug 19;23:101538. doi: 10.1016/j.onehlt.2026.101538 (PMC13545719; doi:10.1016/j.onehlt.2026.101538)
Supplement: Supplementary file 1 [file mmc1.docx]

***Supplementary material on***

**Climatic Drivers of the 2024 Malaria Outbreak in Northeast India: Indirect Role of El Niño**

**Authors:** Avik Kumar Sam, Ujjal Gogoi, Ananta Maji, Arup Borgohain, Rocky Pebam, Tarun Bhatnagar, Keshab Barman, Supriyo Chowdhury, Harish Phuleria*, Ipsita Pal Bhowmick*

*Correspondence: Dr. Ipsita Pal Bhowmick, [bhowmick.ip@icmr.gov.in](mailto:bhowmick.ip@icmr.gov.in); [ipsita.malaria.research@gmail.com](mailto:ipsita.malaria.research@gmail.com), Dr. Harish C. Phuleria, [phuleria@iitb.ac.in](mailto:phuleria@iitb.ac.in)

Figure S1 Granger causality associations for the lagged variables between 2018 and 2024 in (a) Alipurduar and (b) Kokrajhar.

Figure S2 Monthly variation in weather parameters observed at Alipurduar and ENSO anomaly. The red curve shows ENSO anomaly.

Figure S3 Monthly variation in weather parameters observed at Kokrajhar and ENSO anomaly. The red curve shows the ENSO anomaly.

Table S1: Details of Blood Smear Collection (BSC), Blood Smear Examination (BSE), number of cases with *Plasmodium falciparum (Pf),* number of cases with *Plasmodium vivax (Pv)* and Annual Parasite Incidence (API)

| **S. No.** | **Year** | **District** | **Population** | **BSC** | **BSE** | **RDT done** | **Total positive (RDT and/or slide)** | ***Pf*** | ***Pv*** | **Mixed** | **Annual Parasite Incidence (API)** |
| --- | --- | --- | --- | --- | --- | --- | --- | --- | --- | --- | --- |
| 1 | 2019 | Kokrajhar | 991959 | 136953 | 136953 | 90010 | 447 | 219 | 228 | 0 | 4.5 |
| 2 | 2020 |  | 1013450 | 80340 | 80340 | 60502 | 109 | 37 | 72 | 0 | 1.1 |
| 3 | 2021 |  | 1019987 | 72590 | 72590 | 58400 | 32 | 8 | 24 | 0 | 0.3 |
| 4 | 2022 |  | 1041858 | 94788 | 94717 | 48108 | 95 | 29 | 66 | 0 | 0.9 |
| 5 | 2023 |  | 1051813 | 101683 | 101683 | 44800 | 256 | 146 | 110 | 0 | 2.4 |
| 6 | 2024 |  | 1060354 | 239500 | 239500 | 184647 | 3808 | 727 | 2995 | 86 | 35.9 |
| 7 | 2019 | Alipurduar | 1592000 | 636767 | 636767 | NA | 444 | 42 | 386 | 16 | 2.8 |
| 8 | 2020 |  | 1605000 | 88016 | 88016 | 205370 | 43 | 7 | 34 | 2 | 0.3 |
| 9 | 2021 |  | 1639000 | 312081 | 312081 | 69215 | 31 | 3 | 28 | 0 | 0.2 |
| 10 | 2022 |  | 1653000 | 460875 | 456817 | 86231 | 53 | 4 | 39 | 10 | 0.3 |
| 11 | 2023 |  | 1656000 | 465090 | 444184 | 91320 | 41 | 11 | 29 | 1 | 0.2 |
| 12 | 2024 |  | 1661000 | 298288 | 287548 | 170334 | 744 | 23 | 721 | 0 | 4.5 |

NA: Not Applicable
